# Changes in expression of mesothelial *BBS* genes in 2D and 3D after lithium chloride and ammonium sulphate induction of primary cilium disturbance: a pilot study

**DOI:** 10.1007/s43440-023-00513-0

**Published:** 2023-08-04

**Authors:** Erasmia Rouka, Rajesh M. Jagirdar, Ioannis Sarrigeorgiou, Eleanna Pitaraki, Sotirios I. Sinis, Charalambos Varsamas, Eleftherios D. Papazoglou, Ourania S. Kotsiou, Peggy Lymberi, Anastasios Giannou, Chrissi Hatzoglou, Konstantinos I. Gourgoulianis, Sotirios G. Zarogiannis

**Affiliations:** 1 Department of Physiology, Faculty of Medicine, School of Health Sciences, University of Thessaly, BIOPOLIS, 41500 Larissa, Greece; 2 Department of Nursing, School of Health Sciences, University of Thessaly, GAIOPOLIS, 41500 Larissa, Greece; 3 Laboratory of Immunology, Department of Immunology, Hellenic Pasteur Institute, 11521 Athens, Greece; 4 Department of Respiratory Medicine, Faculty of Medicine, School of Health Sciences, University of Thessaly, BIOPOLIS, 41500 Larissa, Greece; 5 Laboratory of Human Pathophysiology, Department of Nursing, School of Health Sciences, University of Thessaly, GAIOPOLIS, 41500 Larissa, Greece; 6 Section of Molecular Immunology and Gastroenterology, I. Department of Medicine, UKE, 20246 Hamburg, Germany; 7 Department of General, Visceral and Thoracic Surgery, University Medical Center Hamburg-Eppendorf, 20246 Hamburg, Germany; 8 Hamburg Center for Translational Immunology (HCTI), University Medical Center Hamburg-Eppendorf, 20246 Hamburg, Germany

**Keywords:** 3D cell cultures, BBSome, Malignant pleural mesothelioma, Mesothelial, Primary Cilium, Tumor spheroids

## Abstract

**Background:**

Malignant pleural mesothelioma (MPM), a rare and aggressive pleural tumor, has significant histological and molecular heterogeneity. Primary Cilium (PC), an organelle of emerging importance in malignancies, has been scarcely investigated in MPM. A critical molecular complex for the PC function is the BBSome and here we aimed at assessing its expression patterns in ordinary 2D and spheroid 3D cell cultures.

**Methods:**

A human benign mesothelial cell line (MeT-5A), MPM cell lines (M14K, epithelioid MPM; MSTO, biphasic MPM), and primary MPM cells (pMPM) were used. Primers specific for the human *BBS1, 2, 4, 5, 7, 9, 18* transcripts were designed, and quantitative real-time PCR (qRT-PCR) was done with *β-actin* as the gene of reference. The relative gene expression across 2D and 3D cultures was analyzed by the expression factor (mean of 1/ΔCt values). With the 2^–∆∆Ct^ method the gene expression fold changes were assessed from qRT-PCR data. Molecular changes using the PC-modulating drugs ammonium sulfate (AS) and lithium chloride (LC) were also determined.

**Results:**

PC was present in all cells used in the study at approximately 15% of the observed area. BBSome transcripts were differentially expressed in different dimensions of cell culture (2D vs. 3D) in all cell lines and pMPM. Treatment with AS and LC affected the expression of the ciliary *BBS2* and *BBS18* genes in the benign as well as in the MPM cells.

**Conclusions:**

These data indicate distinct BBSome molecular profiles in human benign and MPM cells cultured in 2D and 3D dimensions and support the notion that PC genes should be investigated as potential MPM therapeutic targets.

**Supplementary Information:**

The online version contains supplementary material available at 10.1007/s43440-023-00513-0.

## Introduction

Malignant pleural mesothelioma (MPM) is a cancer mainly derived from asbestos exposure with a long latency time of nearly 40 years until diagnosis [1]. The median age at diagnosis is 75 years while the 1- and 3-year survival is 38% and 7% respectively [2]. MPM also depends on the histological subtype since median survival in epithelioid MPM is 19 months, and in biphasic and sarcomatoid 12 and 4 months respectively [3]. In-depth molecular studies have revealed the MPM complexity, showing differences within biphasic MPM, and between pleural and peritoneal malignant mesothelioma at the genomic level [4, 5]. Given its aggressive nature and that it is diagnosed frequently at a very late stage, surgery renders no benefit to patients, and the limited available treatments are chemotherapy (cisplatin with pemetrexed and bevacizumab where licensed) and radiation therapy or palliative care [1]. Occasionally, MPM is also due to genetic predisposition since germline mutations of the BRCA1 Associated Protein 1 (*BAP1*) gene predispose to MPM, and this by itself is a prognostic factor of increased survival for patients under standard chemotherapy [6, 7]. *BAP1* mutations comprise a syndrome, the *BAP1* tumor predisposition syndrome, with an increased risk of developing skin, kidney, eye, and mesothelial tumors [8, 9]. In this context, *BAP1*-inactivated melanocytic tumors have pronounced centrosome amplification with concomitant loss of primary cilium (PC), an organelle of increasing interest in the study of malignancies [10]. *BAP1* gene absence exacerbates the phenotype of PC loss in melanocytic neoplasms, while BAP1 loss is found in nearly half of cases of MPM [9, 10].

Recently PC was detected in patient biopsies of benign pleura and a fraction of epithelioid and biphasic MPM biopsies as evidenced by the immunostaining of a marker of PC, ADP Ribosylation Factor Like GTPase 13B (ARL13B) [11]. In MPM the PC is frequently lost without precluding activation of the Hedgehog (Hh) pathway that is regulated through several activators and suppressors residing on it. One of the important molecular components of the PC is the Bardet-Biedl Syndrome proteins, which form the BBSome (which is a protein octamer comprised of BBS1, BBS2, BBS4, BBS5, BBS7, BBS8, BBS9, BBS18), with critical role in significant cellular functions [12]. The BBSome regulates cargo trafficking to and from the PC including hormone and growth factor receptors, regulates cytoskeletal dynamics and protein turnover, and potentially has a role in gene expression [13]. We previously reported in MPM patients that *BBS1*gene expression is a favorable prognostic factor [14]. Lastly, we recently reported that PC drug-induced perturbation affects both mesothelial and mesothelioma cells, reducing cell viability and migration and variably cell adhesion in 2D, while reducing tumor spheroid formation and collagen matrix invasion in 3D cultures [15].

This study aimed at assessing the BBSome components gene expression levels of benign mesothelial cells (MeT-5A), MPM cell lines (M14K and MSTO; epithelioid and biphasic MPM), and primary MPM cells from an MPM patient. We assessed such differences in 2D as compared to 3D and aimed at identifying whether PC-modulating drugs change BBSome gene expression.

## Materials and methods

### Cell cultures

We used MeT-5A, M14K, and MSTO cell lines (kindly provided by Professor Ioannis Kalomenidis, National Kapodistrian University of Athens). Culture of cells was done in 10% Fetal Bovine Serum (FBS)-RPMI (F0804, Sigma, St Louis, MO, USA) that was supplemented with 2 Mm L-Glutamine (G7513, Sigma), 1% Penicillin/Streptomycin (P4333, Sigma), and 0.5% w/v Plasmocin (ANT-MPP, InvivoGen, Toulouse, France) in a 5% CO_2_ incubator. Cells were synchronized for 24 h in 0.5% FBS-RPMI before experiments. For drug treatments, cells were incubated for 24 h with 30 Mm Ammonium Sulphate (AS; A4915-500G, Sigma), or 50 Mm Lithium Chloride (LC; L4408-100G, Sigma).

To generate 3D spheroids, we employed the hanging drop method [16, 17]. Synchronized cells at a density of 4 × 10^6^ /ml were mixed with 250 ng/mL of sterile bovine plasma fibronectin (341,631-1MG, Sigma) in 10% FBS-RPMI. 25 μL microliter of this cell solution containing 10^5^ cells was used to generate individual spheroids on the underside of sterile petri dishes with 1 ml of sterile PBS in the bottom dish. After 24 h the cell medium was removed with a sterile filter paper and replaced with the appropriate drug treatments for another 24 h. Finally, to collect RNA for gene expression assessments 10 hanging drops were combined and centrifuged at 5000 rpm for 5 min followed by lysis for RNA collection.

### Primary malignant mesothelioma cells (pMPM) isolation from a patient pleural effusion

From a hospitalized MPM patient with pleural effusion in the Department of Respiratory Medicine (University Hospital of Larissa, Greece), 2 Lt of pleural fluid were removed and centrifuged at 1500 rpm for 5 min immediately after drainage. The precipitate was suspended again in 10% FBS-RPMI, washed three times, and plated in 10% FBS-RPMI media for subculturing to use in experiments. Pleural biopsy was acquired using medical thoracoscopy and followed by pathology review [Immunohistochemistry (IHC): positive (+) for Cytokeratin AE1/AE3 (CKAE1/AE3), Vimentin (VIM), WT1 Transcription Factor (WT1), Calretinin; Calbindin 2 (CALB2), and negative (−) BAP-1, Epithelial Cell Adhesion Molecule (EPCAM; MOC31), B72.3, Transcription Termination Factor 1 (TTF1), Platelet And Endothelial Cell Adhesion Molecule 1 (PECAM1; CD31), ETS Transcription Factor ERG (ERG)], that led to a final diagnosis of epithelioid MPM. We obtained informed consent for experimentation from the subject involved. The study was conducted according to the Declaration of Helsinki and was approved by the University Hospital of Larissa Ethics Committee (29,268/16–07-2019).

### Confocal imaging of acetylated tubulin, a surrogate of PC, in cell monolayers

Cytoskeletal buffer (CB) was prepared as follows: 100 mM NaCl (S9888, Sigma), 300 mM sucrose (S0389, Sigma), 3 mM MgCl_2_ (M2670, Sigma), and 10 mM PIPES (P6757, Sigma). The pH was adjusted to 6.9. Subsequently, this solution was warmed to 37 °C and just prior to use, quantities of 250 µL of Triton™ X-100, Tx (T8787, Sigma) and 250 µL of 1 M EGTA (E3889, Sigma) were added in 50 mL of CB. Then cells were washed twice quickly with pre-warmed CB and fixed with a CB/paraformaldehyde mixture for 10 min at 37 °C, thoroughly washed and blocked with 1% Bovine Serum Albumin (BSA)/Phosphate-buffered saline (PBS)/Tx (0.1%) for 1 h at room temperature. After fixation, the cells were incubated with the primary antibody diluted 1:100 (14 mg/mL) in a solution of 1% BSA/PBS/Tx (0.1%) overnight at 4 °C. The primary antibody was acetylated α-tubulin (Lys40) monoclonal antibody (66,200–1-Ig, Proteintech). Extensive washing followed and the cells were incubated in 1:2000 mouse Alexa Fluor 488 secondary antibody (Life Technologies) for 2 h at room temperature and nuclei were labeled with Topro-3. Coverslips were mounted with Mowiol (475,904-M, Sigma). For controls, we performed the same process while omitting the primary antibodies. The preparations were viewed with a 63 × oil objective using a Leica TCS-SP8 confocal microscope (Solmser, Germany), and images were analyzed with ImagePro Plus v5.1. The acquired confocal images (two images per preparation) were analyzed to quantify the density of PC in each cell line using the Fiji extension of ImageJ2 software [18]. This analysis involved the manual counting of PC per cell which was expressed as the percentage of PC-stained cells as a fraction of the total cells per unit area.

### RNA isolation and quantitative real-time PCR assay (qRT-PCR)

For mRNA expression, qRT-PCR was used, and *β-actin* was the gene of reference [17]. We used the NCBI Primer-BLAST tool for the design of primers specific for the human *BBS1, 2, 4, 5, 7, 9, and 18* transcripts (Table 1). Total RNA was isolated in each experimental condition with TRIzol^®^ reagent (15,596–026, Invitrogen). The quality and the quantity of the RNA were assessed using the Quawell Q3000 UV spectrophotometer. Extracted RNA (200 ng) was reverse transcribed into complementary DNA (cDNA) with the SuperScript™ III first-strand synthesis system (18,080,044, Invitrogen™) in a reaction volume of 20 µL. Then cDNA was 1:5 diluted with Ambion™ Nuclease-free water (AM9930, Invitrogen). The diluted cDNA (8.4 μl) and the according primers (0.4 μM) were subsequently added in a 20 μl PCR mix (PowerUp™ SYBR™ Green Master Mix, A25742, Applied Biosystems™) and were then amplified in an ABI 7300 Real-Time PCR System. The thermal cycling conditions in all cases were as follows: 50 °C for 2 min, 95 °C for 2 min followed by 40 cycles of 95 °C for 15 s, 55 °C for 30 s, and 72 °C for 1 min. Three biological replicates were collected from three independent experiments per condition and each of these biological replicates was run in two technical replicates (total N of six).

**Table 1:** Primer pair characteristics of BBSome genes

Gene (symbols)	Primer sequences (5′- > 3′)	Length of product	Template
*BBS1*	Forward: TTTAGCCAAGATGAGCCTTCC Reverse: TGCAGTACTTGGGGTGCTTG	143	NM_024649.5
*BBS2*	Forward: AAACTGCGCCACAAAATCAGC Reverse: GGATTATGAATAAAAACCTTGCCCG	110	NM_001377456.1
*BBS4*	Forward: ATTGGCCTAGGAGATCAGCC Reverse: CAACTGGTCTTGTGCCTTGT	79	NM_001252678.1
*BBS5*	Forward: CTTGTTCACCATGTCGGTGC Reverse: GGTCTTGTTTTCATTTGCTGCG	87	NM_152384.3
*BBS7*	Forward: AGAGGCAGATCACCTACAGGA Reverse: ATCAGTGATCATGCCATAGAGT	76	NM_176824.3
*BBS9*	Forward: TTATTCCAGACCAACAGGCAT Reverse: GCAGTTTTTGAAGGCTGACCT	96	NM_001033605.1
*BBS18*	Forward: GGGATGCATCTTTTCTTCTGTATGC Reverse: CTGCAGCTTTAAGCATCCAACC	83	NM_001195305.3

### Statistical analysis

Comparisons of counted cells with PC and without PC in each cell type assessed via confocal microscopy were performed with the Chi-square test. The relative expression of *BBS* genes is presented using the expression factor (mean of 1/ΔCt values) as previously described [20]. Differences in the expression levels of the PC components between 2 and 3D cell culture systems were assessed by two-way ANOVA with the Tukey multiple comparisons test. Changes in *BBS2* and *BBS18* expression levels associated with drug treatments were assessed with the 2^–∆∆Ct^ method. Log base 2 of 2^–∆∆Ct^ values were used for subsequent analyses with two-way repeated measures ANOVA with Tukey multiple comparisons test. Statistical analyses were done with Prism 9.0 for Windows (San Diego, CA, USA) and values of *p* < 0.05 were considered significant. The data presented are the mean standard error of the mean (SEM).

## Results

### Primary cilia are present in the human mesothelial cell lines and primary mesothelioma cells

**Fig. 1:**
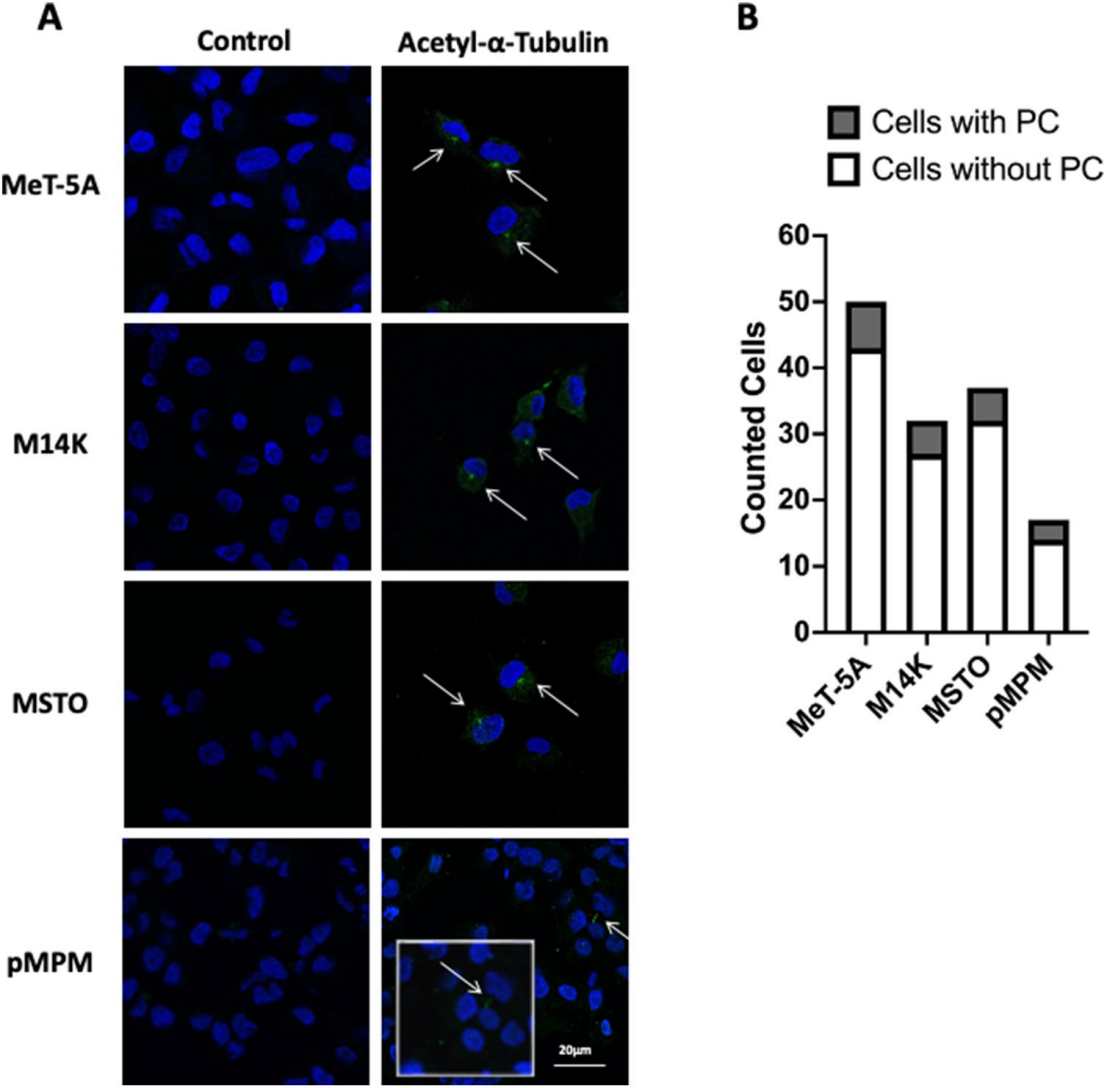
Primary cilium imaging. **A** Primary Cilium indirect immunolabeling of acetylated α-tubulin in MeT-5A benign immortalized mesothelial cells, M14K epithelioid MPM cells, MSTO biphasic MPM and in pMPM primary MPM cells. After fixation, cells were probed for acetylated-α-tubulin (Alexa Fluor 488; green), and nuclear staining (Topro-3, blue). **B** Comparison of counted cells with PC and without PC. Analysis was done by Chi-square test. MeT-5A, M14K and MSTO cells stained positive for PC at a percentage of 14%, 16% and 14% per unit area respectively, whereas for the case of pMPM the percentage was 18%. Control cells were imaged by omitting the primary antibody during the staining process. Scale bars 20 μm

Acetylated α-tubulin is an established surrogate marker of PC in mammalian cells. Thus, we stained acetylated α-tubulin in all cells used and performed confocal microscopy that confirmed the presence of PC in both benign and malignant mesothelial cell lines as well as in pMPM (Fig. 1A; raw images in Supplementary Fig. 1A). We performed image analysis where we quantified the fluorescence intensity due to the acetylated α-tubulin staining and found that PC was evident in 14%, 16%, and 14% of MeT-5A, M14K, and MSTO cells per unit area respectively. PC was evident in 18% of pMPM cells per unit area. The quantification was performed in the images provided in Supplementary Fig. 1B. The comparison of counted cells with PC and without PC among the above cell types (Fig. 1B), showed no significant difference (Chi-square test, χ^2^ = 0.2006, DF = 3, *p* = 0.9775). This result provided evidence that our experimental platform was fit for PC studies.

### BBSome transcripts were differentially expressed in different cell culture model dimensions

**Fig. 2:**
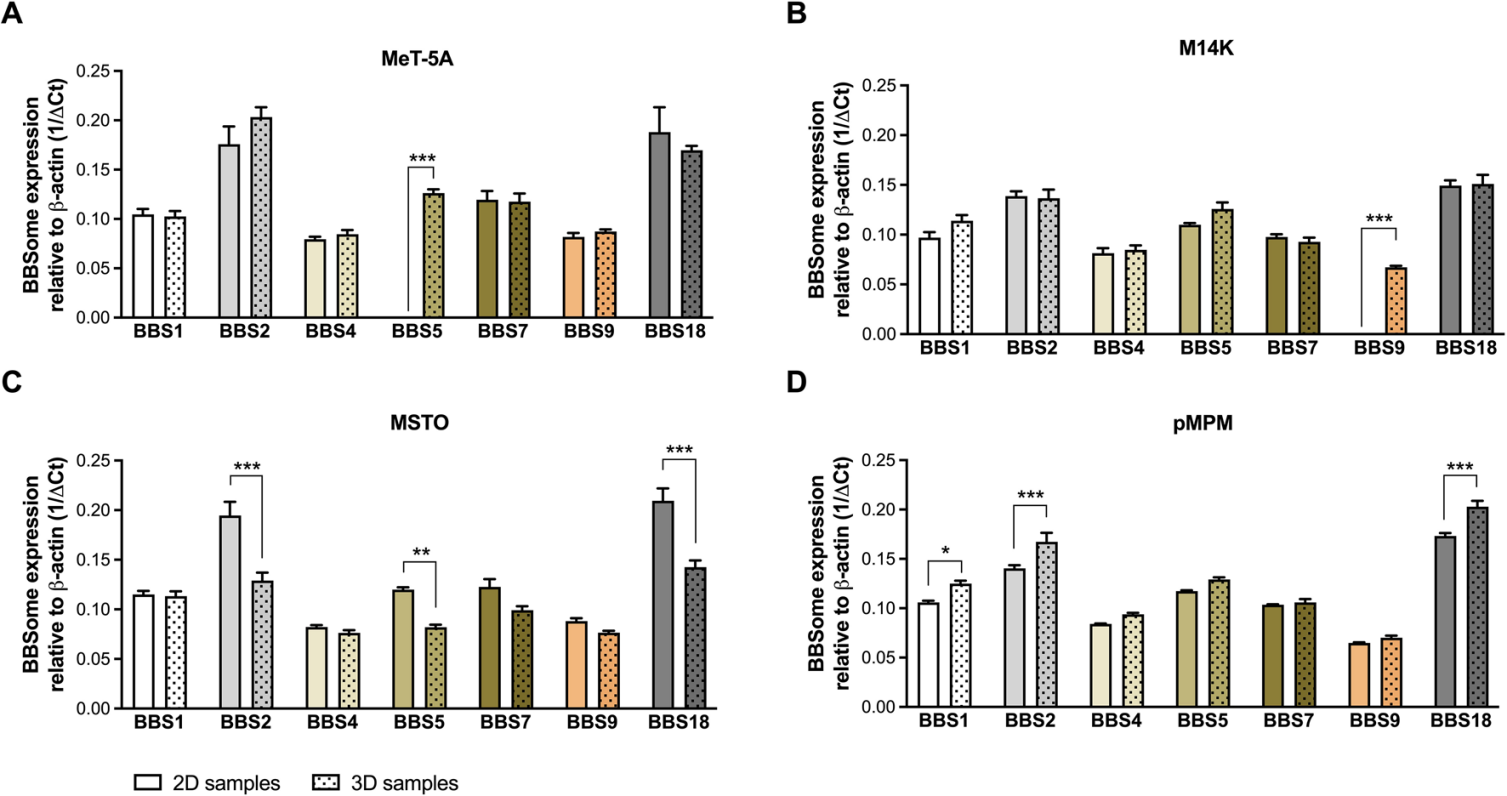
Native BBSome components gene expression. Gene expression levels of primary cilium-associated *BBS* genes, *BBS1*, *BBS2*, *BBS4*, *BBS5*, *BBS7*, *BBS9* and *BBS18*, depending on 2D or 3D culture conditions relative to *β-actin.* **A** MeT-5A benign immortalized mesothelial cells, **B** M14K epithelioid MPM cells, **C** MSTO biphasic MPM cells, **D** pMPM primary MPM cells. N = 3 biological replicates in duplicates in each case. Analysis was done by two-way ANOVA with Tukey multiple comparison test. *p < 0.05, **p < 0.01,***p < 0.001

We observed cell culture system-dependent differences in the expression of *BBS* genes. In MeT-5A cells there was a significant variation in the gene expression patterns of *BBS1, BBS2, BBS4, BBS5, BBS7, BBS9,* and *BBS18* (two-way ANOVA, F_6,70_ = 49.86, *p* < 0.0001) and a significant difference in their expressions between 2 and 3D culture conditions (two-way ANOVA, F_1,70_ = 14.91, *p* = 0.0002). In the multiple comparisons analysis, there was a significant difference in the gene expression of *BBS5* in 3D versus 2D (Tukey multiple comparison test, q = 12.84, DF = 70, *p* < 0.0001) (Fig. 2A).

In M14K cells there was a significant variation in the gene expression patterns of *BBS1, BBS2, BBS4, BBS5, BBS7, BBS9,* and *BBS18* (two-way ANOVA, F_6,70_ = 105.2, *p* < 0.0001) and a significant difference in their expressions between 2 and 3D culture conditions (two-way ANOVA, F_1,70_ = 24.29, *p* < 0.0001). In the multiple comparisons analysis, there was a significant difference in the gene expression of BBS9 in 3D versus 2D (Tukey multiple comparison test, q = 12.63, DF = 70, *p* < 0.0001) (Fig. 2B).

In MSTO cells there was a significant variation in the gene expression patterns of *BBS1, BBS2, BBS4, BBS5, BBS7, BBS9,* and *BBS18* (two-way ANOVA, F_6,70_ = 64.37, *p* < 0.0001) and a significant difference in their expressions between 2 and 3D culture conditions (two-way ANOVA, F_1,70_ = 74.46, *p* < 0.0001). In the multiple comparisons analysis, there were significant differences in the gene expression of BBS2 in 2D versus 3D (Tukey multiple comparison test, q = 9.955, DF = 70, *p* < 0.0001); BBS5 in 2D versus 3D (Tukey multiple comparison test, q = 5.743, DF = 70, *p* = 0.0087); and BBS18 in 2D versus 3D (Tukey multiple comparison test, q = 10.14, DF = 70, *p* < 0.0001) (Fig. 2C).

In pMPM cells, there was a significant variation in the gene expression patterns of *BBS1, BBS2, BBS4, BBS5, BBS7, BBS9,* and *BBS18* (two-way ANOVA, F_6,70_ = 266.8, *p* < 0.0001) and a significant difference in their expressions between 2 and 3D culture conditions (two-way ANOVA, F_1,70_ = 64.12, *p* < 0.0001). In the multiple comparisons analysis, there were significant differences in the gene expression of BBS1 in 3D versus 2D (Tukey multiple comparison test, q = 5.458, DF = 70, *p* = 0.0163); BBS2 in 3D versus 2D (Tukey multiple comparison test, q = 7.695, DF = 70, *p* < 0.0001); and BBS18 in 3D versus 2D (Tukey multiple comparison test, q = 8.457, DF = 70, *p* < 0.0001) (Fig. 2D).

### The expression of the BBS2 and BBS18 genes was significantly altered upon treatment with the PC-modulating drugs AS and LC

Since *BBS2* and *BBS18* were the most abundantly expressed genes in all cell lines used in our study, we opted to assess changes in their expression in both 2D and 3D when the cells were treated with a deciliating agent (AS) or a PC elongating agent (LC).

**Fig. 3:**
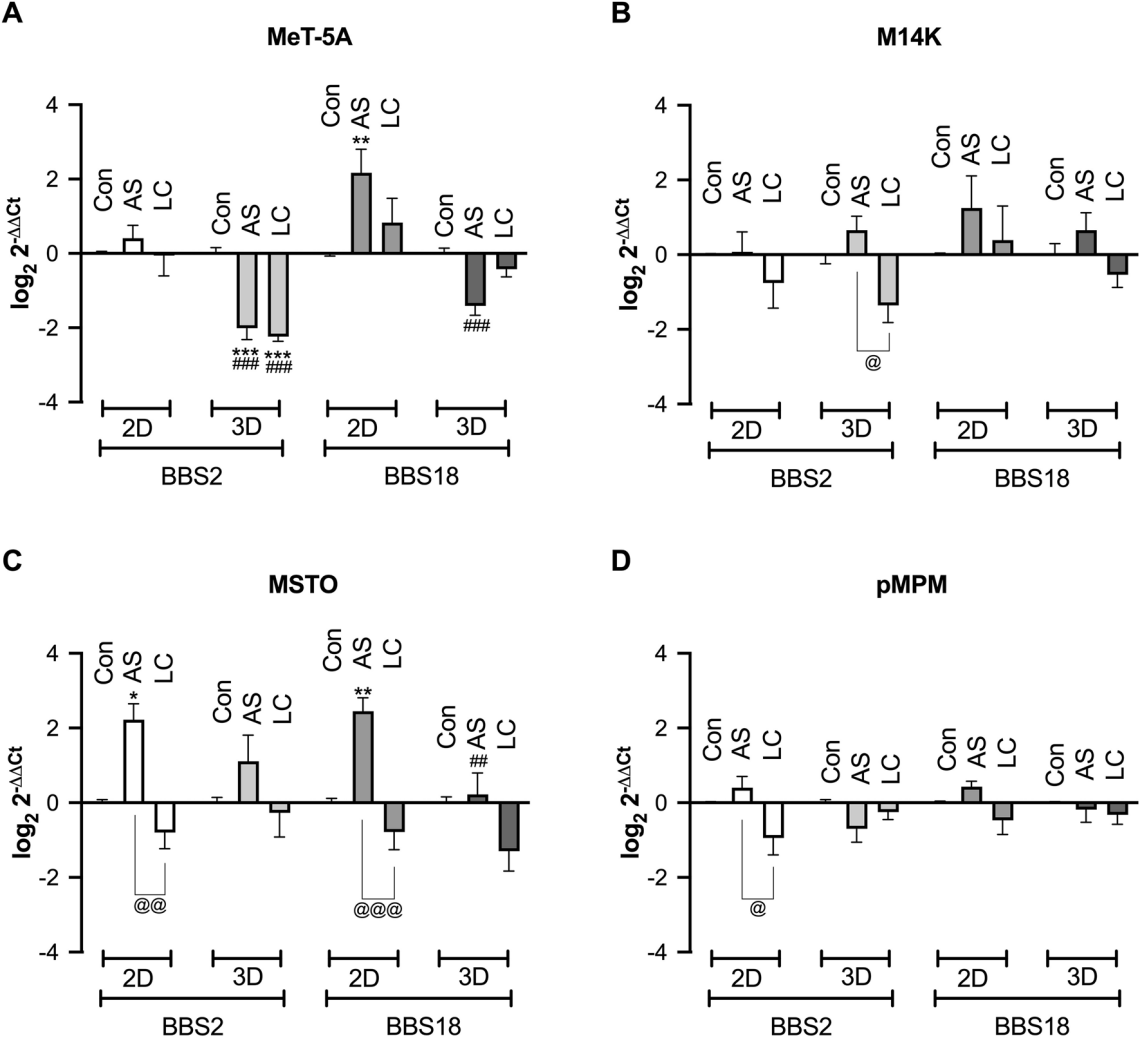
BBSome components gene expression after pharmacological disturbance of the PC. Effect of the primary cilium (PC) modulating drugs, ammonium sulfate (AS; 30 Mm) and lithium chloride (LC; 50 Mm) on the expression levels of the *BBS2* and *BBS18* genes. **A** MeT-5A benign immortalized mesothelial cells, **B** M14K epithelioid MPM cells, **C** MSTO biphasic MPM and **D** pMPM primary MPM cells. N = 3 biological replicates in duplicates in each case. Analysis was done by two-way ANOVA with Tukey multiple comparison test. *p < 0.05, **p < 0.01, ***p < 0.001 vs. Control of the same culture dimension; ^##^*p* < 0.01, ^###^*p* < 0.001 vs PC modulating drugs between the different culture dimensions (2D vs 3D); ^@^*p* < 0.05, ^@@^*p* < 0.01, ^@@@^*p* < 0.001 vs different PC modulating drugs in the same culture dimension (AS vs LC)

Regarding the gene expression of *BBS2* in MeT-5A cells there was a significant variation concerning drug treatments (two-way ANOVA, F_2,30_ = 7.514, *p* = 0.0023) and dimension of culture (two-way ANOVA, F_1,30_ = 38.18, *p* < 0.0001). Especially in 3D spheroid cultures both AS (Tukey multiple comparison test, q = 6.621, DF = 30, *p* = 0.0007) and LC (Tukey multiple comparison test, q = 7.362, DF = 30, *p* = 0.0002) induced the downregulation of the PC *BBS2* gene. Finally, both AS and LC were significantly more potent in 3D cultures compared to 2D (Tukey multiple comparison test, q = 7.971, DF = 30, *p* < 0.0001; q = 7.164, DF = 30, *p* = 0.0003 respectively) (Fig. 3A). As far as the gene expression of *BBS18* in MeT-5A cells is concerned only the aspect of cell culture dimension was significant (two-way ANOVA, F_1,30_ = 24.41, *p* < 0.0001). The AS led to a significant upregulation of *BBS18* in 2D (Tukey multiple comparison test, q = 5.429, DF = 30, *p* = 0.0071) and this differed significantly comparing its effect on 3D (Tukey multiple comparison test, q = 8.961, DF = 30, *p* < 0.0001) (Fig. 3A).

Regarding the gene expression of *BBS2* in M14K cells there was a significant variation only concerning drug treatments (two-way ANOVA, F_2,30_ = 5.684, *p* = 0.0081) as opposed to 2D or 3D culture dimensions. There was also a significant difference between the effect of AS and LC in 3D (Tukey multiple comparison test, q = 4.596, DF = 30, *p* = 0.0309). Regarding the gene expression of *BB18* treatment with either drug in both 2D and 3D cultures resulted in no significant changes (Fig. 3B).

The gene expression of *BBS2* in MSTO cells varied significantly only concerning drug treatments (two-way ANOVA, F_2,30_ = 12.10, *p* = 0.0001) as opposed to 2D or 3D culture dimensions (Fig. 3C). AS treatment as compared to Controls in MSTO cells in 2D resulted in a significant overexpression of *BBS2* (Tukey multiple comparison test, q = 4.76, DF = 30, *p* = 0.0234). There was also a significant difference between the effect of AS and LC in 2D *BBS2* gene expression (Tukey multiple comparison test, q = 6.463, DF = 30, *p* = 0.001). As far as the gene expression of *BBS18* in MSTO cells is concerned there was a significant variation concerning both drug treatments (two-way ANOVA, F_2,30_ = 17.07, *p* < 0.0001) and dimension of culture (two-way ANOVA, F_1,30_ = 7.559, *p* = 0.01). The AS led to a significant upregulation of *BBS18* in 2D (Tukey multiple comparison test, q = 6.007, DF = 30, *p* = 0.0024) and this differed significantly comparing its effect on 3D (Tukey multiple comparison test, q = 5.464, DF = 30, *p* = 0.0067). There was also a significant difference between the effect of AS and LC in 2D *BBS18* gene expression (Tukey multiple comparison test, q = 7.924, DF = 30, *p* < 0.0001) (Fig. 3C).

Finally, in pMPM cells neither the treatment nor the dimension factor altered gene expression levels of *BBS2* and *BBS18* significantly. However, there was a significant difference between the effect of AS and LC in 2D *BBS2* gene expression (Tukey multiple comparison test, q = 4.771, DF = 30, *p* = 0.0229) (Fig. 3D).

## Discussion

This is the first study to assess the gene expression of the PC BBSome in 2D monolayer cultures and 3D spheroid cultures in the context of benign pleural mesothelial and MPM cells. PC has been studied in peritoneal mesothelial cells, where around 40% of cultured cells had PC irrespective of their density [20]. Using the benign mesothelial cell line, MeT-5A, 2 MPM cell lines (M14K and MSTO), and primary MPM cells isolated from a patient with epithelioid MPM we were able to demonstrate the presence of PC in our in vitro system by confocal microscopy. In our experiments, the range of PC positive staining ranged from 14 to 18% depending on the cell line. Indeed, our data corroborate findings on the variable occurrence of PC in human pleural biopsies of inflamed and malignant tissue as well as MPM cell lines [11].

Regarding the BBSome gene expression, we assessed it in 2D and 3D in all three available cell lines and the pMPM cells. In 2D cultures of MeT-5A cells, expression of *BBS1*, *BBS2*, *BBS4*, *BBS7*, *BBS9,* and *BBS18* was detected while notably *BBS5* expression was absent. On the contrary *BBS5* expression in MeT-5A cells was detected in 3D spheroids, along with the expression of *BBS1*, *BBS2*, *BBS4*, *BBS7*, *BBS9,* and *BBS18* that did not differ significantly between the 2D and 3D cultures. This dimension-dependent *BBS5* expression requires further study because BBS4 and BBS5 proteins demonstrate functional redundancy in HEK293 cells. BBSome and a retina-specific *BBS5* splice variant have been reported in mouse retina [21, 22].

Also, a dimension-dependent gene expression difference in the BBSome of M14K cells was found for *BBS9*. Tumor Protein P53 (TP53), a tumor suppressor, activates *BBS9* transcription to promote ciliogenesis and differentiation of human embryonic stem cells [23]. *TP53* is frequently mutated in MPMs and experimental evidence shows that its inactivation drives MPM development [24]. The absence of expression of *BBS9* in 2D as opposed to 3D could be attributed to changes in TP53 expression although in ovarian and endometrial cancer such expression changes were not demonstrated histologically [25]. In MSTO MPM cells, significant differences in *BBS2*, *BBS5,* and *BBS18* gene expression were found and, in all cases, the expression was significantly lower in 3D spheroid cultures. Conversely, in pMPM the expression of *BBS1*, *BBS2,* and *BBS18* was significantly higher in the 3D cultures. MPM cells are amongst the most versatile human cells functionally and morphologically, while being heterogeneous at the inter-tumor as well as the intra-tumor level, as established through histological and molecular analyses [26]. A recent study has identified two distinct epithelioid MPM subtypes regarding gene expression, while the PC status in MPM tissues has demonstrated significant variability [11, 27]. Thus, the use of 3D cultures would be ideal given the changes in MPM behavior concerning ECM and tissue architecture [16, 17]. Overall, a biological explanation for the *BBS* gene differences that we have observed would relate to the fact that the mitotic spindle assembly checkpoint (MSAC) pathway is one of the most deregulated pathways in MPM and thus the microtubular network that is highly associated with the PC is a point of pathophysiological and therapeutic interest in pleural malignancy [28]. Considering that the BBSome is the point of convergence of the microtubular stability and ciliogenesis, the variation that we observe in our results regarding the differential expression of BBSome genes between benign and MPM cells is expected [29]. On the other hand, cell shape and nucleus morphology reflect complex genomic and gene expression changes in cancer cells that associate with cell motility and mechanical behavior, especially during metastasis and cancer progression [30–32]. PC has a role in cell migration and spherogenesis in MPM and since dimensionality affects cell morphology the differences in *BBS* gene expression patterns between 2 and 3D cultures could further be explained by such biological phenomena [15]. Based on the above it would be reasonable to pursue studies that will demonstrate that gene silencing of differentially expressed *BBS* genes would affect 2D cell migration and 3D spherogenesis.

Among all the BBS genes that were assessed the ones that were highly expressed were *BBS2* and *BBS18*. Another important finding of our study was that the expression levels of the *BBS2* and *BBS18* genes change upon treatment with PC modulators. AS triggers cell deciliation [33, 34], while LC induces PC elongation [35, 36]. *BBS2* and *BBS18* have not so far been implicated in any studies relative to cancer, so the relevance of our findings cannot be compared with the published literature. *BBS2* gene expression in MeT-5A cells was significantly reduced in 3D after the effect of AS and LC, and it was unchanged in M14K and pMPM cells, while in MSTO cells AS significantly increased its expression in 2D. *BBS18* gene expression was sensitive to the effects of AS that significantly increased it in 2D in MeT-5A, and MSTO cells, and in 3D its effect was significantly decreased in MeT-5A cells as compared to 2D.

Considering the involvement of PC in cancer signaling pathways such as Extracellular signal-regulated kinase (ERK)/Mitogen‑activated protein kinase (MAPK), Hedgehog (Hh), and Wnt, further investigation is warranted concerning its role and therapeutic potential in MPM [37]. Also, in a recent study, we showed that mesothelial PC disturbance by AS and LC has effects is many cancers related phenotypes such as cell migration, spheroid formation and invasion, and mesothelial to mesenchymal potential and therefore future studies should focus on the specific role of each differentially expressed *BBS* gene in these phenotypes [15]. A limitation of our study was that we did not provide data on BBS proteins expression since technical difficulties in western blots have been reported previously [19]. Despite these constraints, our study describes for the first time the *BBS* molecular components of PC in benign and MPM grown both in 2D and 3D environments.

Here we provided evidence for a differential expression of BBSome components in different MPM histotypes and after their treatment with PC-modulating drugs in 2D and 3D. *BBS2* and *BBS18* gene expression were the most pronounced in all cell lines tested. Building on this finding future experiments will need to assess whether the gene expression of *BBS2* and *BBS18* changes under certain stimuli that are relevant to malignancy, such as oxidative stress, inflammation, and osmotic imbalance to name a few [38–40]. Furthermore, in the same line of thinking assessing how their gene expression patterns change under the influence of pleural effusions of different etiologies will provide more in-depth information on the role of BBSome in the context of pleural diseases in general. These experiments will need to include gene silencing and overexpression to dissect the exact contribution of each BBSome component on MPM pathobiology and potential therapeutics. Finally, demonstrating that expression of BBSome components at the protein level in biopsies of MPM patients using IHC will strengthen the potential of BBSome and PC targeting as a mean of therapy in MPM.

## Conclusions

In this study, confocal image analysis confirmed the presence of PC in benign and malignant mesothelial cells. Molecular investigation showed that the relative expression of PC-associated *BBS* genes depends on in vitro cell culture dimensionality and is sensitive to PC drug modulators. The provided data highlight the significance of 3D models used in molecular studies of MPM and support the hypothesis that PC should be further investigated as a therapeutic target.

### Supplementary Information

Below is the link to the electronic supplementary material.

Supplementary file1 (PPTX 14603 KB)

## Data Availability

Upon reasonable request, the data are deemed available from the corresponding author.

## Abbreviations

2D: 2 Dimensions
3D: 3 Dimensions
ARL13B: ADP Ribosylation Factor Like GTPase 13B
AS: Ammonium Sulfate
BAP1: BRCA1 Associated Protein 1
BBS: Bardet-Biedl Syndrome
BSA: Bovine Serum Albumin
CALB2: Calbindin 2
CKAE1/AE3: Cytokeratin AE1/AE3
cDNA: Complementary DNA
CB: Cytoskeletal buffer
FBS: Fetal Bovine Serum
EPCAM: Epithelial Cell Adhesion Molecule
ERG: ETS Transcription Factor ERG
ERK: Extracellular signal-regulated kinase
Hh: Hedgehog
IHC: Immunohistochemistry
MAPK: Mitogen‑activated protein kinase
LC: Lithium Chloride
MPM: Malignant Pleural Mesothelioma
μL: Microliter
PBS: Phosphate-buffered saline
PC: Primary Cilium
PECAM1: Platelet And Endothelial Cell Adhesion Molecule 1
pMPM: Primary Malignant Pleural Mesothelioma
qRT-PCR: Quantitative Real-Time Polymerase Chain Reaction
SEM: Standard Error of the Mean
TP53: Tumor Protein P53
TTF1: Transcription Termination Factor 1
VIM: Vimentin
WT1: Wilms Tumor 1
