# Supplementary material for: Changes in expression of mesothelial BBS genes in 2D and 3D after lithium chloride and ammonium sulphate induction of primary cilium disturbance: a pilot study
Source: Pharmacol Rep. 2023 Aug 4;75(5):1230–9. doi: 10.1007/s43440-023-00513-0 (PMC10539424; doi:10.1007/s43440-023-00513-0)
Supplement: Supplementary file 1 — Supplementary file1 (PPTX 14603 KB) [file 43440_2023_513_MOESM1_ESM.pptx]

## Slide 1
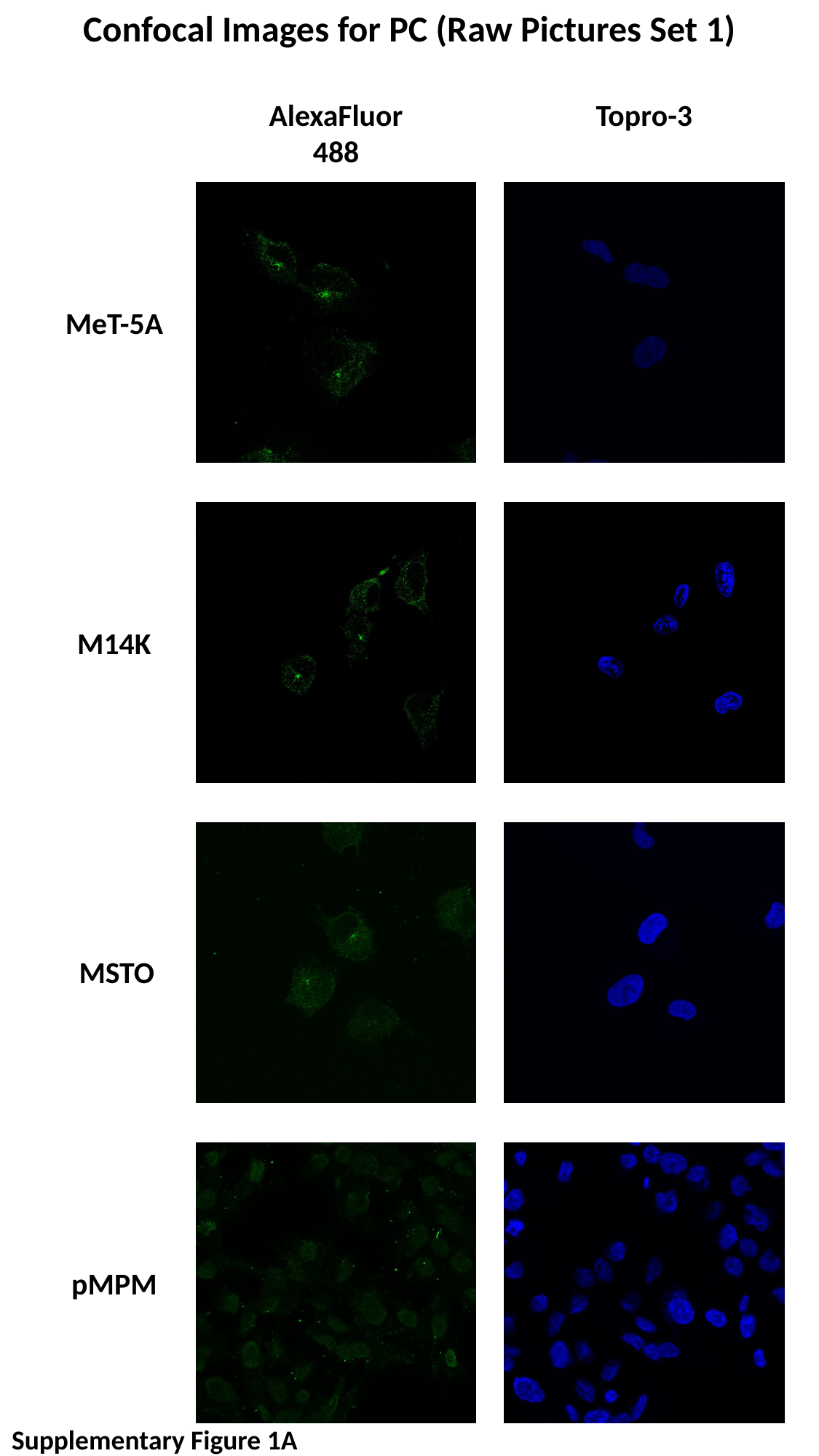

Confocal Images for PC (Raw Pictures Set 1)
Topro-3
AlexaFluor 488
MeT-5A
M14K
MSTO
pMPM
Supplementary Figure 1A

## Slide 2
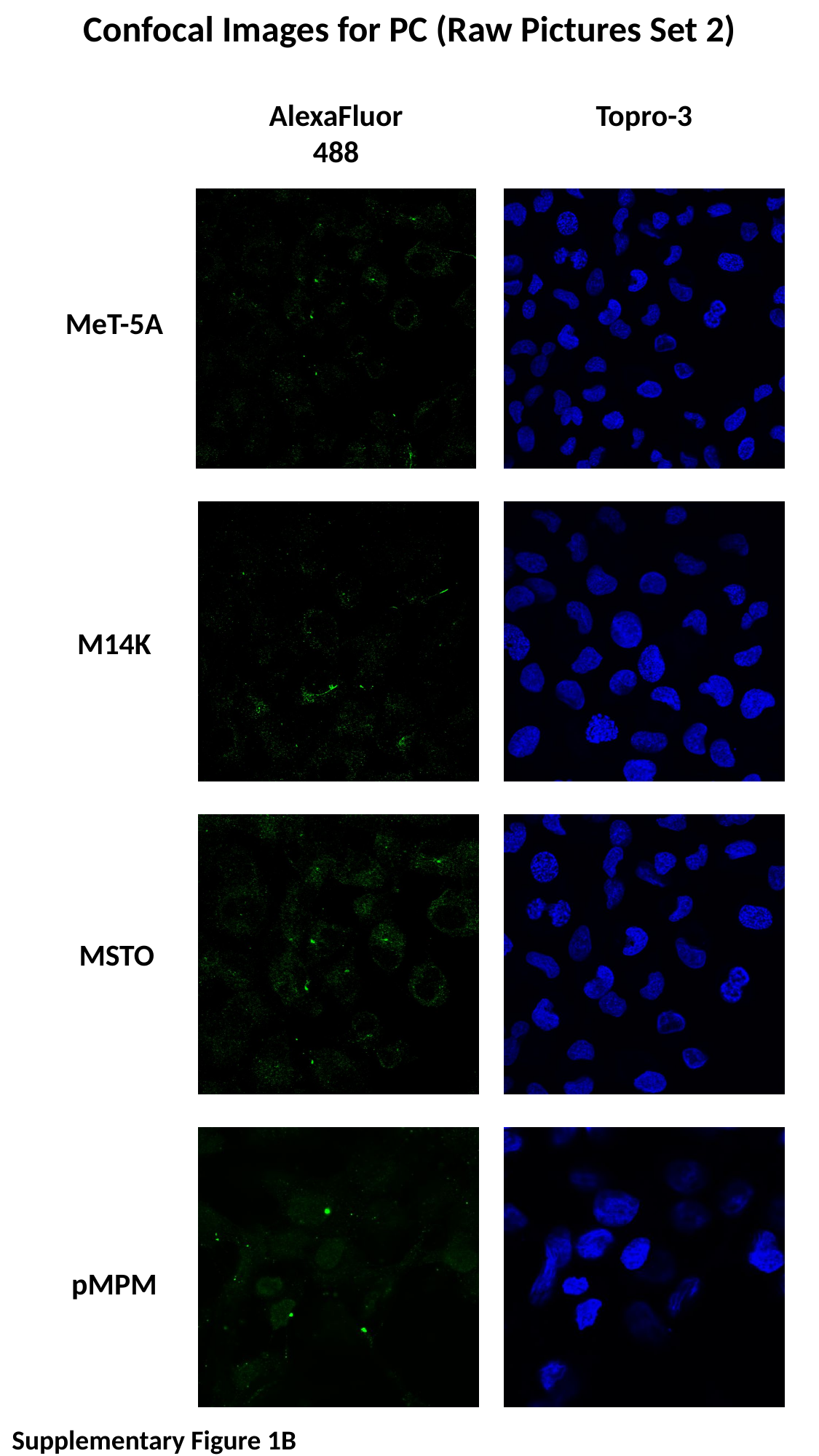

Confocal Images for PC (Raw Pictures Set 2)
Topro-3
AlexaFluor 488
MeT-5A
M14K
MSTO
pMPM
Supplementary Figure 1B
